# Stochastic Dynamic Aircraft System Conflict Distribution under Uncertainties

**DOI:** 10.3390/e24050583

**Published:** 2022-04-21

**Authors:** Anrieta Dudoit, Vytautas Rimša, Marijonas Bogdevičius

**Affiliations:** 1 Department of Aviation Technologies, Vilnius Gediminas Technical University (VILNIUS TECH), LT-10223 Vilnius, Lithuania; vytautas.rimsa@vilniustech.lt; 2 Department of Mobile Machinery and Railway Transport, Vilnius Gediminas Technical University (VILNIUS TECH), LT-10223 Vilnius, Lithuania; marijonas.bogdevicius@vilniustech.lt

**Keywords:** air traffic management system, dynamic aircraft system, conflict, stochastic distribution, random parameters, wind

## Abstract

A dynamic aircraft system conflict (concurrent event) situation exists when a time with a loss (-es) of separation (LOS) in their true or predicted trajectories is determined. Regional air traffic management (ATM) programs aim to make ATM safer and more efficient through a higher level of automation for such processes as dynamic aircraft systems concurrent events detection and, consequently, resolution. Therefore, wind and aircraft speed uncertainty parameters should be properly addressed. This paper offers an approach to a dynamic aircraft system flying under a certain concurrent event situation and demonstrates situation stochastic distribution results (output) based on determined wind speed values (while wind direction angles and the dynamic aircraft system speed values are random). Based on these facts, the stochastic dynamic aircraft system conflict distribution information under determined and random parameters might be retrieved at any specific (preferred) time moment. The observations of this study disclosed that such stochastic output data might have a certain impact on safety matters (potential “domino effect” conflicts on a horizontal plane) and on the efficiency (i.e., flight distance which eventually is a determinant of flight time, fuel costs, delays, emissions, monitoring, etc.).

## 1. Introduction

A challenging aspect of the dynamic aircraft system safe and efficient flights is to detect and resolve conflict (concurrent event) situations. Since the European Air Traffic Management (ATM) system needs modifications as it is under considerable stress and since air traffic is predicted to grow significantly by 2040 in comparison with 2019 air traffic levels, this challenging aspect becomes even more demanding [1].

As some steps to make the current ATM system safer and more efficient while accommodating predicted traffic growth were taken in perspective of the Single European Sky (SES) ATM Research (SESAR) program, the aim of which is to develop and implement the future ATM system via free route airspace (FRA) with 4D trajectories incorporation within the European Functional Airspace Blocks (FABs) though the major concerns of a such transition should take into account such variety of aspects as uncertainties such as wind, aircraft speed, temperature, navigation and human errors, passenger delays, etc.

The purpose of this paper is to analyze the dynamic aircraft system conflict point determination and its distribution in the ATM system under uncertainties, since the analysis of the determined wind speed and random wind direction angles, unstable aircraft speed impact on a certain dynamic aircraft system trajectories configuration conflict points determination and its distribution (same time unit) was identified as the approach which needs further focus. In this way, stochastic-like conflict detection and, consequently, conflict resolution (the latter is beyond the scope of investigation of this paper) could be further expanded and may supplement the works of another authors.

## 2. Literature Review

A dynamic aircraft system determined conflict point is described as two aircraft being in conflict (concurrent event) situation at a time, *t*, with a loss of separation (LOS) (i.e., which is violation(s) of the minimum separation criteria between two aircraft trajectories) in their true or predicted trajectories [2,3]. However, such conflicts indicate uncertainties (i.e., the dependance among the random variables like wind and a dynamic aircraft system speed for a certain aircraft flight trajectories configuration, should be analyzed due to its stochastic (random) distribution).

It was discovered that some efforts were made in the past by other authors to analyze the problem of conflict detection under the presence of uncertainties (i.e., wind, etc.). Such uncertainty related analyses were mainly conducted in terms of conflict detection and resolution [4,5,6] as conflict intensity and probability [7,8,9,10,11,12,13,14,15,16,17,18] involving accuracy or optimal path planning [19,20], or trajectory uncertainty [21,22] and efficiency [23] of aircraft trajectory prediction and synthesis [24], fuel consumptions [25], time management prior to take off with the aim of aircraft conflict situations deconfliction [26,27,28,29], etc.

Most of the conflict detection and resolution algorithms are mainly divided into two main categories: deterministic and probabilistic approaches, which are divided into the relevant sub-categories [4,7]. In this paper the probabilistic conflict detection approach is taken for more deeper analysis in respect to the deterministic method. Probabilistic conflict detection regarding wind uncertainty on aircraft motion model could be described by using the empirical distribution model of future aircraft positions [4,30], the dynamic model by using stochastic differential equations [31] and the probabilistic aircraft model based on the hybrid systems [16] and conflict probability between aircraft could be estimated for detection of potential conflicts [8].

**Figure 1:**
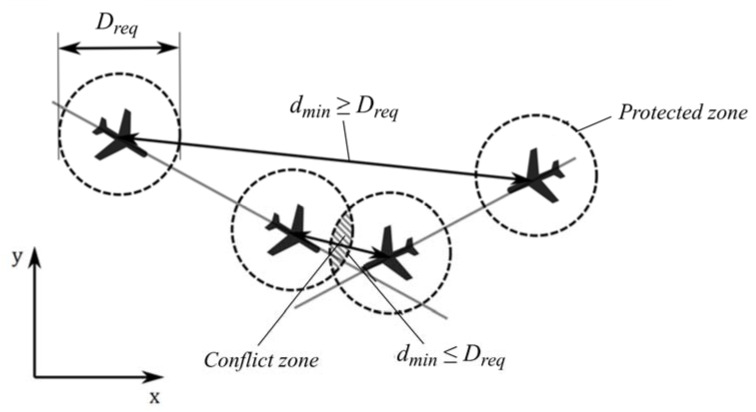
Two aircraft conflict situation on a horizontal plane [8].

A violation of a given set of separation minima can be discovered by a conflict search, i.e., computation and comparison of the predicted flight paths of two or more aircraft where their protected zones touch or overlap (Figure 1).

Conflict for two aircraft flying at the same level on a conflicting course exists when the minimum separation $d_{min}$ between them is equal or smaller than the minimum required safe horizontal separation $D_{req}$, i.e., $d_{min}\leq D_{req}$ (Figure 1). The minimum required safe horizontal separation in the analyzed airspace is 5 nm between a dynamic aircraft system [32,33].

Detection of a conflict is one of the main ATM system functions used to ensure air traffic safety and efficiency [15] and is a point from which the proposal to avoid such conflict begins. The deconfliction method and time to commence resolution maneuvers depend on the probability of a collision. Determining the probability of a conflict shall be based on the position and velocity of an aircraft [34] considering uncertainty in the future location of the aircraft, caused by wind, imprecision in navigation and operation of the aircraft [30,35]. The important thing is that the earlier we obtain a prediction, the less confident it is [36]. According to Kuchar and Yang [4] conflict detection is the process of deciding ‘*when*’ the action should be taken, and conflict resolution means ‘*how*’ or ‘*what*’ action should be taken.

Conflict detection is one of an important responsibilities of the air traffic controller job and no doubt one of the most complex. Some indicators and appropriate metrics to quantify conflict situations were previously defined and are classified as: conflict intensity and conflict probability. Such indicators deliver sufficient information about conflicts and could be used by air traffic controllers (ATCOs) for better decision making [8].

However the free flight concept assumes that all airspace users will plan their preferred routes freely [37], therefore the consequence of this will be an increase in the number of intersection points of the flight trajectories, i.e., conflicts, called ‘Blind spots’ which are hard to be identified since they are not “standard” or “fixed routes” hotspots. As Eurocontrol Network Manager Operational Safety Review [38] states for the period of a 6 year study: 36% of the European severity A and B sample of incidents for the years 2015–2020 involved a conflict generated by “Blind spot” where ATCO overlooked a potentially conflicting proximate aircraft when clearing or instructing another one. Thus such a ‘chaotic’ organization of ATM system and predicted increase of traffic levels, would increase the number of potential conflicts also. Moreover, considering the fact that such conflict points are affected by uncertainties as wind and aircraft speed, especially at high levels, this should be properly addressed in terms of safety and the cost-effectiveness.

The remainder of this paper is arranged as follows. First, in Section 2 the literature review is presented. In Section 3, a stochastic dynamic system conflict point distribution under wind and the dynamic aircraft system speed influence is described. In Section 4, simplified investigation of a stochastic conflict point distribution under wind uncertainties is presented. The validation of a stochastic model is demonstrated in Section 5. The discussion of our results is presented in Section 6. Finally, some conclusions and observations are drawn in Section 7.

## 3. Stochastic Aircraft Conflict Distribution under Uncertainties

**Figure 2:**
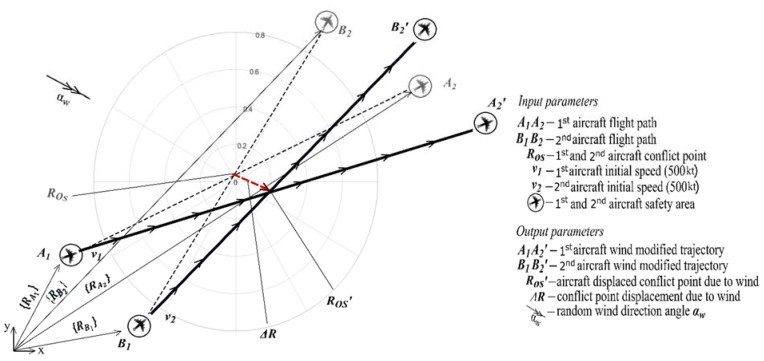
Aircraft conflict point displacement due to uncertainties.

This paper proposes an approach to the dynamic aircraft system (i.e., two aircraft in a level flight), taking into account determined and random parameters effect on a stochastic parameters distribution for a certain configuration of the dynamic aircraft system (angle between the flight trajectories and distance to a conflict point) involved in a conflict situation when such the dynamic aircraft system flight distance from the intersection point and flight speed are such that the time of appearance at such a conflict point is the same. Using such data, we could define aircraft trajectories relevant information (new conflict points locations and their displacement angles, etc.) under a certain determined wind speed parameters at a specific (preferred) time unit. This situation of wind impact on the conflict point of a dynamic aircraft system with random speed values is schematically presented in Figure 2.

An example of a conflict situation as was introduced in [39] is described as follows:

1. Simulation start points: one aircraft is in point $A_{1}$ with coordinates $\left(x_{1},y_{1},z\right)$ while the other aircraft is in point $B_{1}$ with coordinates $\left(x_{3},y_{3},z\right)$.
2. Simulation end points: $A_{2}\left(x_{2},y_{2},z\right)$ and $B_{2}\left(x_{4},y_{4},z\right)$, determine the flight direction of aircraft, respectively.
3. It is assumed that both aircraft are in the same Air Traffic Control (ATC) sector and that a minimum separation of 5 nm applies in the examined airspace.
4. The problem in two dimensions is analyzed and it is assumed that the aircraft fly at the same flight level, which means that a vertical separation of 1000 ft is not applied. Therefore, in further calculations, the vertical coordinate *z* is omittted.
5. A dynamic aircraft system time of occurence at a conflict point is the same.
6. The determined and the stochastic (random) parameters could differ.

The starting and endpoints of both aircraft are represented through vectors {$R_{A_{1}}$}, $\{R_{A_{2}}$}, $\left\{R_{B_{1}}\right\}$ and $\left\{R_{B_{2}}\right\}$ (Figure 2). The trajectories of both aircraft are denoted by

(1) $$T_{A}=\{R_{A_{1},A_{2}}\}=\{R_{A_{2}}\}-\{R_{A_{1}}\},$$

(2) $$T_{B}=\{R_{B_{1},B_{2}}\}=\{R_{B_{2}}\}-\{R_{B_{1}}\},$$

where

(3) $$\left\{R_{A_{1}}^{T}\right\}=\left[x_{1},y_{1}\right],\;\{R_{A_{2}}^{T}\}=\left[x_{2},y_{2}\right]\;,\{R_{B_{1}}^{T}\}=\left[x_{3},y_{3}\right],\;\{R_{B_{2}}^{T}\}=\left[x_{4},y_{4}\right]$$

The coordinates of the point $R_{O_{s}}$, where the trajectories $T_{A}$ and $T_{B}$ intersect i.e., at the determined dynamic aircraft system conflict point (without the uncertainty) are defined as follows [40]:

(4) $$R_{O_{s}}=\left[\begin{array}{c} \frac{\left(x_{1}y_{2}-y_{1}x_{2}\right)\left(x_{3}-x_{4}\right)-\left(x_{1}-x_{2}\right)\left(x_{3}y_{4}-y_{3}x_{4}\right)}{\left(x_{1}-x_{2}\right)\left(y_{3}-y_{4}\right)-\left(y_{1}-y_{2}\right)\left(x_{3}-x_{4}\right)}\\ \frac{\left(x_{1}y_{2}-y_{1}x_{2}\right)\left(y_{3}-y_{4}\right)-\left(y_{1}-y_{2}\right)\left(x_{3}y_{4}-y_{3}x_{4}\right)}{\left(x_{1}-x_{2}\right)\left(y_{3}-y_{4}\right)-\left(y_{1}-y_{2}\right)\left(x_{3}-x_{4}\right)} \end{array}\right]$$

It is evident that if the aircraft speeds and aircraft trajectories distance to intersection point $R_{O_{s}}$, are the same, aircraft would reach a certain point, in this case intersection point $R_{O_{s}}$ at the same time unit $T_{OS}$:

(5) $$T_{OS}=L_{1}/v_{1}=L_{2}/v_{2},$$

In such a case from the intersecting point $R_{O_{s}}$, we move backwards towards simulation start time vectors {$R_{A_{1}}$} and {$R_{B_{1}}$} in this way ensuring that the conflict of two aircraft would appear at the intersection point {$R_{O_{s}}$} at the same time unit $T_{OS}$. Thus the dynamic (two aircraft) system intersection condition could be expressed as follows:

(6) $$L_{1}=\frac{v_{1}}{v_{2}}L_{2},$$

Speed relationship/ratio Equation (7) is expressed with the help of coefficient ɑ as follows:

(7) $$\begin{array}{c} ɑ=\frac{v_{1}}{v_{2}},\\ L_{1}=ɑL_{2}. \end{array}$$

The crossing point of two aircraft is analysed taking into account wind direction and its speed, when first aircraft speed is $v_{1}$ and second aircraft speed is $v_{2}$. Furthermore, initial aircraft speed are the same i.e., $v_{1}\;$= $v_{2}$, though they are random during the simulation, i.e., $v_{1}\neq v_{2}$. where:

$L_{1}$ and $L_{2}$—distances of the dynamic aircraft system appearance at the same time from intersection point $\left\{R_{O_{s}}\right\}$ to the initial simulation points $\{R_{A1}$} and {$R_{B1}\}$, respectively.

First and second aircraft vectors coordinates at any time moment *t* could be determined from Equations (8) and (9):

(8) $$R_{1}\left(t\right)=R_{A1}+e_{\tau 1}v_{1}t+e_{N1}\left({\left\{e_{W}\right\}}^{T}\left\{e_{N1}\right\}v_{W}\right)t=R_{A1}+c_{1}t,$$

(9) $$R_{2}\left(t\right)=R_{B1}+e_{\tau 2}v_{2}t+e_{N2}\left({\left\{e_{W}\right\}}^{T}\left\{e_{N2}\right\}v_{W}\right)t=R_{B1}+c_{2}t,$$

where:

$e_{\tau 1}$ and $e_{\tau 2}$—unit vectors, which coincide with the dynamic aircraft system flight directions, i.e., $e_{\tau 1}$ coincides with a trajectory $T_{A}$ and $e_{\tau 2}$ coincides with a trajectory $\;T_{B}$, as described in the Equations (10) and (11):
  (10) $$e_{\tau 1}=\left(R_{A2}-R_{A1}\right)/\left|R_{A2}-R_{A1}\right|$$
  (11) $$e_{\tau 2}=\left(R_{B2}-R_{B1}\right)/\left|R_{B2}-R_{B1}\right|$$

where:

$R_{A1},R_{A2}$—first aircraft trajectory initial and final vectors determined from the beginning of two-dimensional Cartesian coordinate system;
$R_{B1},R_{B2}$—second aircraft trajectory initial and final vectors determined from the beginning of two-dimensional Cartesian coordinate system;
$e_{N1}$ and $e_{N2}$—unit vectors, which direction is perpendicular to unit vectors $e_{\tau 1}$ and $e_{\tau 2}$ vectors and to the dynamic (both aircraft) system flight directions, i.e., $T_{A}$ and $T_{B}$.
  (12) $$e_{N1}=e_{z}\times e_{\tau 1},\;e_{N2}=e_{z}\times e_{\tau 2},$$
$c_{1}$ and $c_{2}$—vectors as described in Equations (13) and (14).
  (13) $$c_{1}=e_{\tau 1}v_{1}+e_{N1}\left({\left\{e_{W}\right\}}^{T}\left\{e_{N1}\right\}v_{W}\right),$$
  (14) $$c_{2}=e_{\tau 2}v_{2}+e_{N2}\left({\left\{e_{W}\right\}}^{T}\left\{e_{N2}\right\}v_{W}\right),$$

where:

${\left\{e_{W}\right\}}^{T}=\left[\begin{array}{ccc} \cos\left(\alpha \right) & \sin\left(\alpha \right) & 0 \end{array}\right]$—wind direction unit vector;
$v_{W}$—wind speed (taken as a determined parameter),
$\alpha_{w}$—the angle between the wind direction vector $e_{W}$ and the X (0°) axis.

It is assumed that when the dynamic aircraft system trajectories intersect, both of a dynamic aircraft system trajectories intersection point coordinates vectors are equal, i.e., $R_{1}\left(t_{OS}\right)=R_{2}\left(t_{OS}\right)$. This means that applying Equations (8) and (9), we might define flight time $T_{OS}$ Equation (15) determined as a time to the new stochastically dispalaced trajectories intersection point $R_{O_{s}}^{\prime }$ due to uncertainties, like wind direction angles and the dynamic aircraft speed analyzed for the certain configuration of the trajectories:

(15) $$t_{OS}={\left\{c_{1}\right\}}^{T}\left(\left\{\left|R_{O_{s}}^{\prime }\right|\right\}-\left\{R_{A1}\right\}\right)/\left({\left\{c_{1}\right\}}^{T}\left\{c_{1}\right\}\right),$$

where: $R_{O_{s}}^{\prime }$—the new stochastically distributed dynamic aircraft system flight trajectories intersection point, i.e., conflict point, which could be defined from the equation system as presented in Equation (16):

(16) $$\begin{array}{c} \left[A\right]\left\{{R_{OS}}^{\prime }\right\}=\left(\left\{R_{B1}\right\}-\left[A_{0}\right]\left\{R_{A1}\right\}\right),\\ \left[A_{0}\right]=\left\{c_{2}\right\}{\left\{c_{1}\right\}}^{T}/\left({\left\{c_{1}\right\}}^{T}\left\{c_{1}\right\}\right);\;\left[A\right]=\left(\left[I\right]-\left[A_{0}\right]\right); \end{array}$$

[I]—identity matrix.

Wind and the dynamic aircraft system speed influence on intersection point $R_{O_{s}}^{\prime }$ is defined by the distance Δ*R* which could be described as the displacement from the dynamic aircraft system initial trajectories intersection point (when wind speed is equal to zero $\left\{R_{OSW}\left(v_{W}=0\right)\right\}$ to a new displaced intersection point due to a determined wind speed parameter {${R^{\prime }}_{OSW}\left(v_{W}\neq 0\right)\}$. This displacement is presented as follows:

(17) $$\Delta R=\sqrt{{\left\{\Delta R_{OSW}\right\}}^{T}\left\{\Delta R_{OSW}\right\}},$$

where: {$\Delta R_{OSW}\}=\left\{R_{OSW}\left(v_{W}=0\right)\right\}-\left\{{R^{\prime }}_{OSW}\left(v_{W}\neq 0\right)\right\}$.

The direction of vector {$\Delta R_{OSW}$} is expressed by angle φ in relation to X (0°) reference axis and could be defined from Equations (18) and (19):

(18) $$cos\;\varphi ={\left\{e_{\tau 1}\right\}}^{T}\left|\Delta R_{OSW}\right|/\left|\Delta R_{OSW}\right|,$$

(19) $$\varphi =arccos({\left\{e_{\tau 1}\right\}}^{T}\left|\Delta R_{OSW}\right|/\left|\Delta R_{OSW}\right|).$$

The parameters in Formulas (1)–(19) are used in a simplified investigation of a stochastic dynamic aircraft system conflict point and its stochastic distribution under uncertainties, and the relevant results are presented in the Section 5. Moreover, this method can be applied when the wind direction and speed can change over time.

## 4. Simplified Investigation of a Stochastic Conflict Point Distribution under Uncertainties

Using the concept of presented methodology in Section 3 of this paper, the three cases of the stochastic examined dynamic aircraft system conflict points stochastic distributions under uncertainties like wind and random aircraft speed influence for a certain aircraft trajectories configuration are compared. The comparison is presented using an example with determined coordinates of examined dynamic aircraft system as from [39]. The coordinates of the simulation start and end points (expressed in nautical miles) are as follows:

(20) $$R_{A_{1}}^{T}=\left[30,41,z\right]\;R_{A_{2}}^{T}=\left[70,59,z\right]\;R_{B_{1}}^{T}=\left[40,34,z\right]\;R_{B_{2}}^{T}=\left[61,68,z\right]$$

The initial mean value of the dynamic aircraft system (i.e., the two middle category aircraft), speed is $v_{1}=v_{2}=500\;\mathrm{kt}$ [41]. The initial conflict point coordinates ($R_{O_{s}}$), regarding initial aircraft speed ($v_{1}=v_{2})$ and wind ($v_{w}=$ 0) values, are calculated from (20) and are as follows:

(21) $$R_{O_{s}}^{T}=\left[49.84,49.92,z\right]$$

The reference axis is X which value is 0°. The first aircraft trajectory $T_{1}$ angle with X (0°) axis amounts 25° and the angle between both aircraft trajectories, i.e., $T_{1}$ and $T_{2}$, is approx 34°.

Direction of measurement of wind is from X (0°) axis and is counterclockwise.

### 4.1. Wind Uncertainty

Wind is one of the influencing and uncertain factors on ATM related problems, and eventually on aircraft flight trajectory evolution, mainly on the dynamic aircraft system conflict point, which could be generated by winds, or which could be influenced/modified/transformed by prevailing winds [28]. So as the wind is the most important source of uncertainty in the trajectory configuration on a horizontal plane as due to the inaccuracy of the forecasts, such assumptions must be addressed: (1) wind uniformity throughout the trajectories during a simulation, and (2) the variety of wind regarding different geographical locations [28].

**Figure 3:**
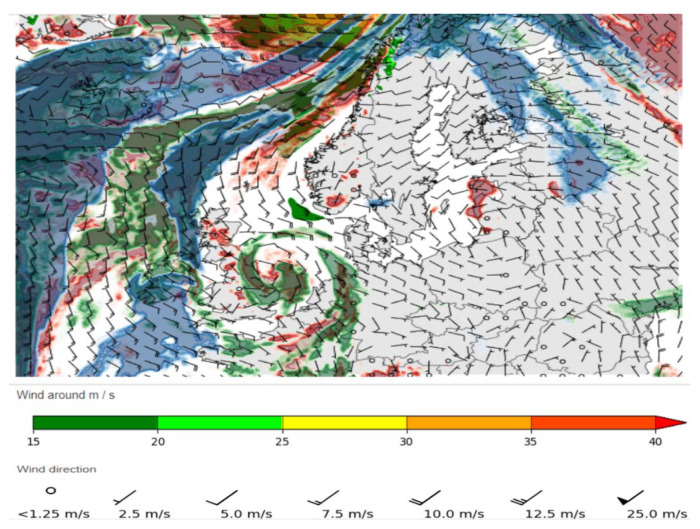
A generic scheme of wind velocity (direction and speed) (see: www.lennuilm.ee, accessed on 3 March 2022).

As wind is taken as a uniform parameter for every direction measured from X (0°) axis and expressed in 5, 10, 15, 20, 25 m/s, i.e., 10, 20, 30, 40, 50 kt, respectively, wind direction angle mean value is taken as $\mu_{\alpha }$ = 0° and wind direction angles vary by a standard deviation $\delta_{\alpha }$ as is analyzed in three cases of this paper and with random aircraft speed and aircraft flight trajectories layout/configuration—these parameters have the specific impact on conflict points stochastic distribution. Nonetheless depending on different flight trajectories layout/configurations these wind and speed parameters would have a different affect. These wind values are taken for similar wind velocity (direction and speed) conditions for the European region. The generic scheme is presented in Figure 3 [42].

The assumption of this study is that wind uncertainty influences a dynamic aircraft system flight paths/trajectories regarding the nominal flight path i.e., without the wind.

### 4.2. Stochastic Conflict Point Distribution under Uncertainties

A conflict point ($R_{O_{s}}$) of a dynamic aircraft system when influenced by wind ($v_{w}$ ≠ 0) is transposed/shifted to a new conflict point ($R_{O_{s}}^{\prime }$) depending on several factors such as wind direction angle ($\alpha_{w})$ and its speed ($v_{w})$, dynamic aircraft system speed ($v_{1}\neq v_{2})$ and an angle between trajectories, etc.

The simplified investigation results of an examined dynamic aircraft system for a stochastic distribution of its conflict point ($R_{O_{s}}^{\prime }$) for ($v_{w}\neq \;0)$ and the relevant parameter (Δ*R)*, in respect to the conflict point ($R_{O_{s}}$) for ($v_{w}$ = 0), are presented and assumptions are partially taken according to previous papers, i.e.,

(1) *Determined* wind speed ($v_{w}\neq \;0)$ *parameter* values are: 5, 10, 15, 20, 25 m/s, i.e., 10, 20, 30, 40, 50 kt, respectively.
(2) *Random* parameters (which are spread according to a normal distribution) are:
  - wind direction angle ($\alpha_{w})$;
  - 1st and 2nd aircraft speed ($v_{1})$ and ($v_{2}).$
(3) *Relevant (output) parameter* is Δ*R*—displacement from initial conflict situation ($R_{O_{s}}$) for ($v_{w}$ = 0) to random conflict point ($R_{O_{s}}^{\prime }$) when ($v_{w}\neq \;0)$.

For simulation to be more close to a realistic case −5 × 10^6^ simulation points were used, i.e., for wind angle $\alpha_{w}$ = 100; for 1st aircraft speed $v_{1}$ = 500 and 2nd aircraft speed $v_{2}$ = 100 simulation points. We have a cycle of 3 random parameters, i.e., $\alpha_{w}$, $v_{1}$ and $v_{2}$ which comprise 5 × 10^6^ of possible combinations, i.e., simulation points which are stochastically distributed as demonstrated in *Case 1*, *Case 2*, and *Case 3.*

### 4.3. 3 Cases of Conflict Point Distribution under Uncertainties

The *Case 1*, *Case 2*, and *Case 3* deal with the situation where the dynamic aircraft system conflict point and its distribution due to determined ($v_{w}$ ≠ 0) and random ($\alpha_{w}$, $v_{1}$ and $v_{2}$) parameters (Table 1), is a stochastic one.

*Case 1*—represents stochastic dynamic aircraft system conflict points distribution under uncertainties such as when determined wind speed parameter is $v_{w}$ ≠ 0 and such a distribution is presented for: $v_{w}$ = 5 m/s (green field), $v_{w}$ = 10 m/s (violet field), $v_{w}$ = 15 m/s (yellow field), $v_{w}$ = 20 m/s (orange field) and $v_{w}$ = 25 m/s (blue field). Meanwhile the random parameters as wind direction angle with mean value $\mu_{\alpha_{w}}$= 0° coincides with X axis, and its standard deviation is $\delta_{\alpha_{w}}=\pm$13° (in total approx ± 39°), while both aircraft mean speed value is $\mu_{v_{1}}$ *=* $\mu_{v_{2}}\;$*=* 500 kt and its standard deviation amounts $\delta_{v_{1}}$*=* $\delta_{v_{2}}\;$= ±6 kt (Figure 1).

*Case 2*—represents stochastic dynamic aircraft system conflict points distribution under uncertainties such as when determined wind speed parameter is $v_{w}$ ≠ 0 and amounts as in *Case 1*. M Meanwhile the random parameters as wind direction angle with mean value $\mu_{\alpha_{w}}$= 0° coincides with X axis, and its standard deviation is $\delta_{\alpha_{w}}=\pm$20° (in total approx ±60°), while both aircraft mean speed value is $\mu_{v_{1}}$ *=* $\mu_{v_{2}}\;$*=* 500 kt and its standard deviation amounts $\delta_{v_{1}}$= $\delta_{v_{2}}$= ±6 kt (Figure 1).

*Case 3*—represents stochastic dynamic aircraft system conflict points distribution under uncertainties such as when determined wind speed parameter is $v_{w}$ ≠ 0 and amounts as in *Case 1.* Meanwhile the random parameters as wind direction angle with mean value $\mu_{\alpha_{w}}$= 0° coincides with X axis, and its standard deviation is $\delta_{\alpha_{w}}=\pm$26° (in total approx ± 78°), while both aircraft mean speed value is $\mu_{v_{1}}$ *=* $\mu_{v_{2}}\;$*=* 500 kt and its standard deviation amounts $\delta_{v_{1}}$= $\delta_{v_{2}}$= ±6 kt (Figure 1).

**Figure 4:**
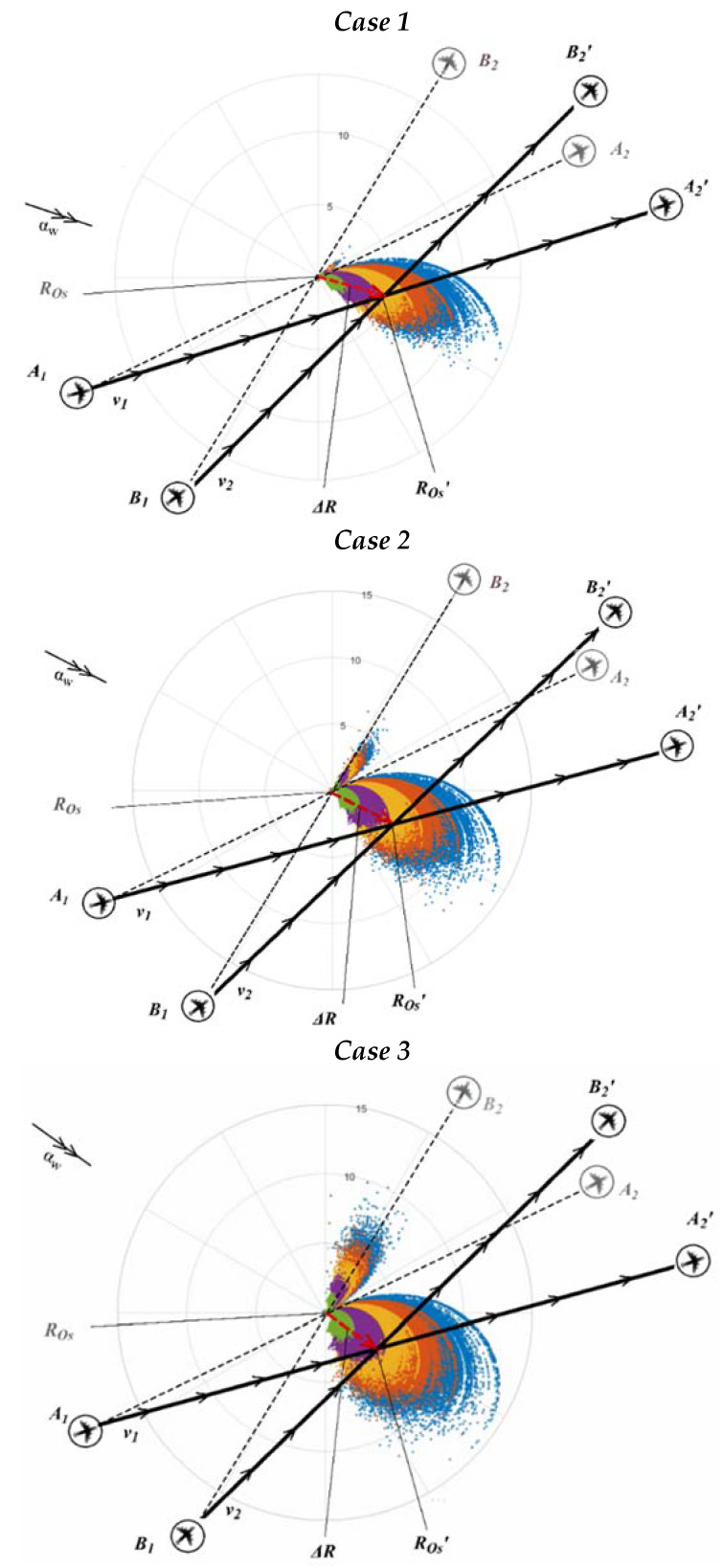
Aircraft conflict point distributions under uncertainties (*Case 1*, *Case 2*, and *Case 3*).

A dynamic aircraft system conflict point ($R_{O_{s}}^{\prime }$) and its stochastic distribution due to wind ($v_{w}$ ≠ 0) from nominal conflict point ($R_{O_{s}}$) varies in respect to wind and a dynamic aircraft system speed uncertainty, as demonstrated below (Figure 4).

It is obvious from the dynamic aircraft system conflict point distribution presented in *Case 1*, *Case 2*, and *Case 3* (Figure 4), that depending on the determined wind speed $v_{w}$ values (0–25 m/s), random wind angles ($\alpha_{w})$ (for a certain wind direction angle deviation, i.e., $\delta_{\alpha_{w}}\;$= ±13°, ±20° and ±26° determined from mean value $\mu_{\alpha }$ *=* 0°) and 1st and 2nd aircraft speed (for a certain standard speed deviation, i.e., $\delta_{v}$ = ±6 kt), the aircraft conflict point stochastic distribution is significant when wind is the most severe and its standard deviation from mean value is the greatest and when it blows perpendicular to aircraft directions of flight (trajectories $T_{1}$ and $T_{2}),$ i.e., when aircraft experience a crosswind than when wind blows from behind both aircraft and in between their flight directions, while the smallest displacement of a dynamic aircraft system, i.e., conflict point distribution, is observed when 1st aircraft experiences a tailwind. *Case 1* demonstrates the smallest stochastic conflict point distribution in comparison to *Case 2* and *Case 3* due to the narrowest of the three cases of wind direction angle ($\alpha_{w})$ standard deviation ($\delta_{\alpha_{w}})$ spectra (dispersion) determined from mean value $\mu_{\alpha }$ *=* 0°. The *max* conflict point displacement Δ*R* (*Case 1*, *Case 2*, and *Case 3*) from initial conflict situation ($R_{O_{s}}$) values are provided in Table 2.

Data values from Table 2 expose that the more severe the wind ($v_{w}$) and the wider wind dispersity, the greater the displacement Δ*R* values of a dynamic aircraft system from initial conflict situation ($R_{O_{s}}$), which could be taken into account when calculating aircraft flight distances and relevant time in respect to a nominal reference (FPL) trajectory or a desired (when wind results in a shorter flying time) trajectory and as a consequence in estimations of fuel costs and emissions. It is evident, that $\Delta R_{max}$ ratio for *Case 1* amounts 16%, for *Case 2*—17%, and for *Case 3*—18%. Moreso, such *max* displacement Δ*R* values with the rest of sufficient data ($\alpha_{w},\;v_{1},\;v_{2}$, φ, etc.) could be used to establish each new (random) conflict point coordinates and in respect to that the appropriate and timely conflict resolution maneuvers could be applied or aircraft speed might be modified so as to ensure a deconflicting situation with a radius of 5 nm around each of newly (randomly) identified conflict point coordinates.

Moreover, the importance of the subtraction results of two pairs of cases (*Case 1* Δ*R* distribution for $\delta_{\alpha_{w}}$ = ±13° and *Case 3* Δ*R* distribution for $\delta_{\alpha_{w}}$= ±26°) and (*Case 1* Δ*R* distribution for $\delta_{\alpha_{w}}$ = ±13° and *Case 2* Δ*R* distribution for $\delta_{\alpha_{w}}$= ±20°) is valuable to analyze them in terms of safety and efficiency.

During subtraction process of two pairs of cases the obtained result showed that the greater residue left for a pair *Case 1* and *Case 3* amounts 3.1 × 10^5^/5 × 10^6^ of population points for all wind speed values, which is approximately 6% of all population points, while for a pair *Case 1* and *Case 2* subtraction case, the residue of such stochastically distributed points amounts 2.7%. This means that such percentage left after subtraction processes taking into account uncertainties, like wind and random dynamic aircraft speed, have an impact on aircraft position which consequently may lead to a “horizontal domino effect” situation (i.e., a secondary conflict with the third aircraft) and may cause undesirable inefficiency in terms of distance, time, costs, emissions, conflict situation monitoring, etc. This should not be neglected by ATC or conflict detection systems when dealing with conflict (concurrent event) situations.

### 4.4. Probability Density Function

**Figure 5:**
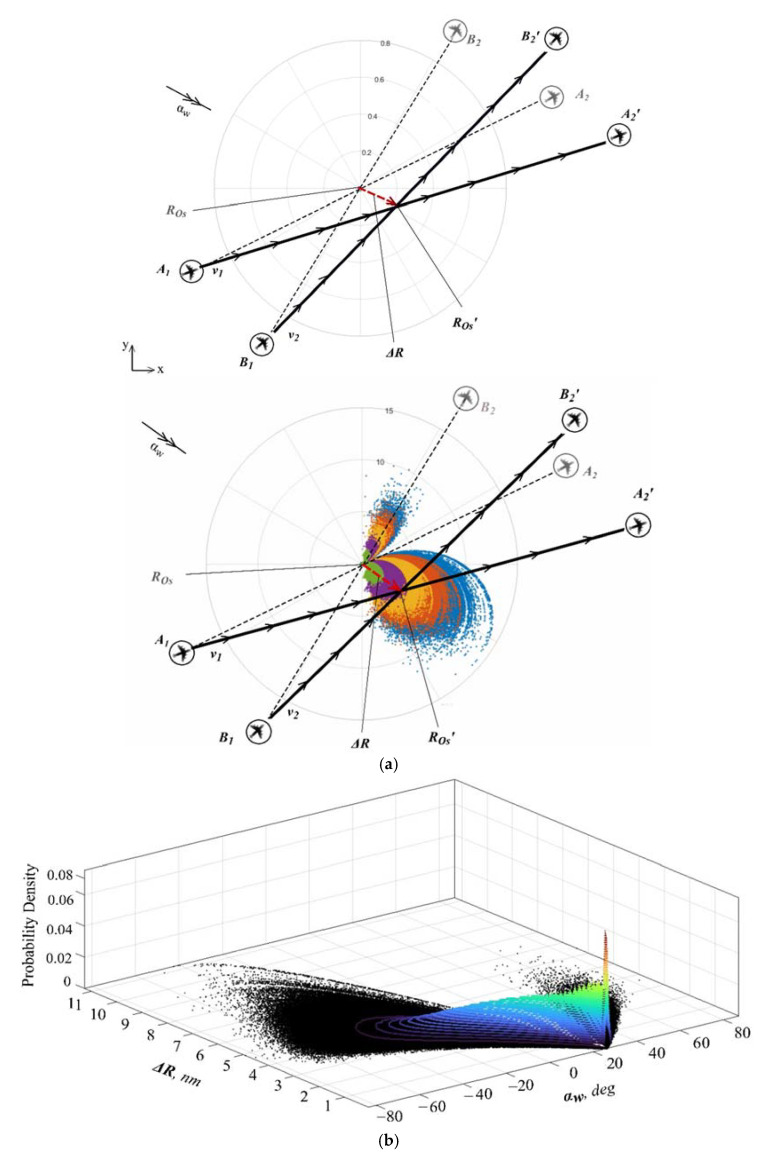
Conflict point under uncertainties and *Case 3* (**a**) and its probability density function (**b**) for wind speed $v_{w}=25\;m/s$.

For estimation of conflict point probability, a probability density function (PDF) is used which describes the probability of the value of a continuous random variable falling within a range [43]. Thus, such PDF could be a good indicator to determine the number of simulation conflict points belonging to specific random variables $\alpha_{w}$ and Δ*R* combination range (area) as generated by the certain determined ($v_{w})$ and random parameters ($\alpha_{w}$, $v_{1}\;\;\mathrm{and}$ $v_{2}$) collocation and is graphically presented in Figure 5 below.

As it is evident from Figure 5b for random variables $\alpha_{w}$ = 26° (where the crosswind changes into the tailwind) and Δ*R* ≈ 0.8 nm combination within the range (area) when $v_{w}=25\;m/s$ (conflict points distribution for variable $\alpha_{w}$ = 26° presented in a blue colour in Figure 5b), the PDF amounts 0.06 ≈ 6% of all 5 × 10^6^ simulation points, which is approximately 3 × 10^5^ of all simulation points. This can be explained as when both aircraft experience a transition from a crosswind to a tailwind the PDF reaches the highest value due to the combination of determined and random variables (parameters).

## 5. Validation of a Stochastic Model

**Figure 6:**
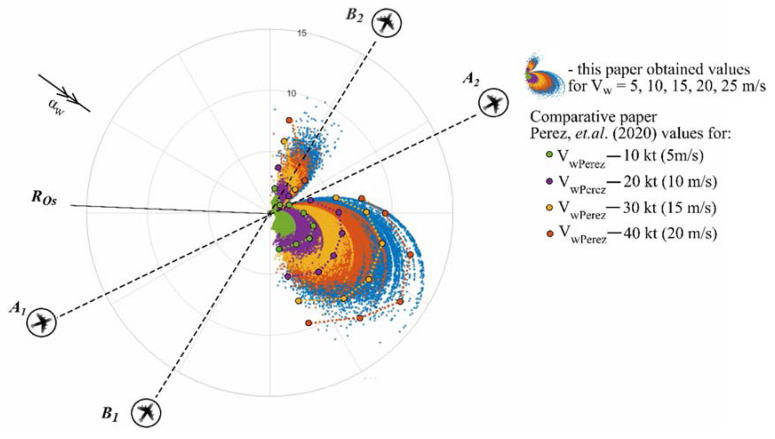
The comparison of the results.

**Figure 7:**
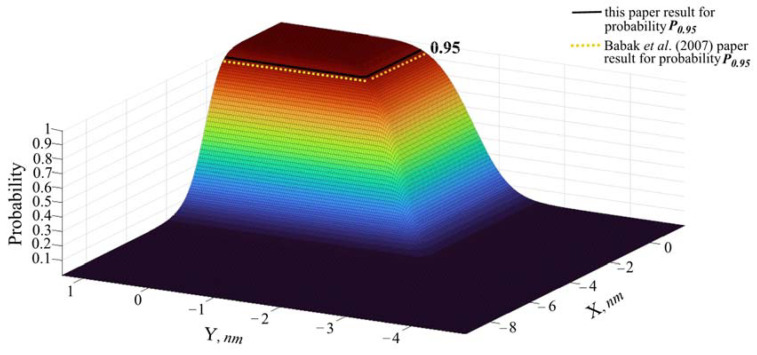
The comparison of conflict point results.

The following numerical results of the simplified study obtained in this article on the stochastic conflict point and its distribution under wind and aircraft speed uncertainties were compared with rearranged according to our criteria model of Perez et al. [28], which analyzed wind impact on the conflict point in terms of departure time allocation prior to aircraft take-off (Figure 6) and an additional comparison with the stochastic model of Babak et al. [17] was made (Figure 7).

While comparing this paper simplified mathematical model results with the rearranged according to our criteria Perez et al. [28] model results, the calculations clearly showed that this paper simplified mathematical model results are more accurate and amount approximately $v_{w}\;\approx \;0.7\;v_{w_{\mathrm{Perez}}}$, this difference of results may be influenced by the initial conditions and modelling parameters i.e., mean values of $\alpha_{w}$, $v_{1}\;,v_{2}$ and they standard deviations $(\mu_{\alpha_{w}}$, $\mu_{v_{1}}$ and $\mu_{v_{2}}$, respectively), and aircraft flight trajectories configuration, and permits to state that the set of the parameters used in this paper allows to realize the conflict point distribution under uncertainties more accurately, which is very important in aviation in terms of safety and effectiveness. Moreso, the results of Perez et al. [28] and this paper results for the upper stochastically spread fields of conflict points show similar distribution.

For the second comparison of this paper stochastic model the numerical solution of conflict probability of random variables combination is obtained and presented in Figure 6 through the cumulative distribution function (CDF), which statistically means a function whose value is the probability that a corresponding continuous random variable has a value less than or equal to the argument of the function, i.e., the cumulative distribution function (CDF) calculates the cumulative probability for a given *x*-value [44]. The CDF for a discrete random variable is defined as

(22) $$F_{x}\left(x\right)=P\;\left(X\;\leq \;x\right)$$

where:

*X*—the probability that takes a value equal to or less than *x* and it lies between the interval (*a*, *b*], *a* < *b*, i.e., 0 and 1.
*x*—a random variable.

Hence, the probability *P* with the interval is given by:

(23) $$P\;(a<X\leq b)=F_{x}\left(b\right)-F_{x}\left(a\right)$$

Based on the second comparison results of stochastic model conflict point with Babak et al. [17] authors stochastic model the numerical solution of random variables combination probability for value of *P* = 0.95 is obtained and illustrated graphically in Figure 7 through the CDF.

Figure 7 demonstrates that this paper proposed stochastic model of conflict point Cumulative Distribution Function for random variables combination probability for value of *P* = 0.95 was obtained by its division to the total area, is slightly smaller than in case of Babak et al. [17] model and the overall probability ratio $P_{ratio}$ ≈ 0.9 Babak et al. [17] value, i.e., this paper stochastic model probability ratio of the area with probability *P* = 0.95 to the total area amounts $P_{ratio}=6\;\mathrm{nm}/60\;\mathrm{nm}=0.1$ and Babak et al. [17] stochastic model probability ratio amounts $P_{ratio}=3.7\;\mathrm{nm}/33.3\;\mathrm{nm}=0.11$, respectively. 

## 6. Discussion

The stochastic dynamic aircraft system conflict distributions analysis presented in this paper is based on the consideration of wind and such a dynamic aircraft system speed as a source of uncertainty since the wind magnitude and direction and related both aircraft speed will make a significant encounter on the flight trajectories thus on dynamic aircraft system conflict points. From the initial coordinates the whole stochastic model was produced, and simulation results were obtained. Moreover, the proposed method could be applied when the wind direction and speed can change over time.

While applying the PDF for a certain determined and random parameters collocation it was revealed that for random variables $\alpha_{w}$ = 25° and Δ*R* ≈ 0.8 nm combination within the range (area) when $v_{w}=25\;m/s$ the PDF amounts 0.06 ≈ 6% of all 5 × 10^6^ simulation points, which is approximately 3 × 10^5^ of all simulation points. This can be explained as when both aircraft experience a transition from a crosswind to a tailwind the PDF reaches the highest value due to the combination of determined and random variables (parameters).

During a first comparison of the proposed stochastic model results with Perez et al. [28] authors results it was discovered that the produced method in this paper allowed to achieve better results mainly due to the fact that the initial conditions and modelling parameters are better which eventually has effect on smaller chance of secondary conflict with a third aircraft and deviation from nominal flight path, which eventually is a determinant of flight time, fuel costs, delays, emissions, monitoring, etc.

Comparing this paper obtained CDF results with a stochastic model of Babak et al. [17] it was identified that the produced method in this paper allowed us to achieve slightly smaller random variables combination probability for value 0.95 and the probability ratio $P_{ratio}$ ≈ 0.9 Babak, et al. [17] results.

As this stochastic dynamic aircraft system conflict distribution model allowed us to achieve quite satisfying results it can further be improved including integration and application of the appropriate conflict resolution algorithms.

## 7. Conclusions

In presented paper the dynamic aircraft system (i.e., two aircraft, conflict point and its stochastic distribution under uncertainties, such as wind and random dynamic aircraft system speed, was studied). The simplified investigation of a proposed model/approach and the obtained initial results disclosed the following:

(1) Through the literature research it was identified that some efforts were made in the past by other authors to analyze the problem of conflict detection under the presence of uncertainties (i.e., wind, aircraft operations, navigation errors, etc.). However, the purpose of this paper was to analyze uncertainties on the dynamic aircraft system conflict point and its distribution in the ATM system since the analysis of the determined wind speed and random wind direction angles, unstable aircraft speed impact on a certain dynamic aircraft system trajectories configuration conflict point, and its distribution was identified as the approach which needs additional focus. As a result, this paper’s results could be expanded and may supplement the works of other authors.
(2) During the subtraction process of two pairs of cases, the obtained result showed that the greater residue is left for a pair *Case 1* and *Case 3* amounts 3.1 × 10^5^/5×10^6^ of population points for all wind speed values, which is approximately 6% of all population points, while for *Case 1* and *Case 2* subtraction case, the residue of such stochastically distributed points amounts 2.7%. This means that such percentage left after subtraction processes taking into account uncertainties consequently could have an impact on a “horizontal domino effect” situation, i.e., to a secondary conflict with the third aircraft and cause undesirable inefficiency in terms of distance, time, costs, emissions, etc. Moreover, PDF reaches its highest value at $\alpha_{w}$ = 26° and is bigger close to it due to the transition from crosswind to tailwind area.
(3) While validating our results with other authors’ analyses (rearranged according to our data), we found out that our mathematical model is more accurate up to approximately $v_{w}\;\approx \;0.7\;v_{w_{\mathrm{Perez}}}$. This difference of the comparison results may be influenced by the initial conditions and modelling parameters, i.e., mean values of $\alpha_{w}$, $v_{1}\;,v_{2}$ and they standard deviations $(\mu_{\alpha_{w}}$, $\mu_{v_{1}}$ and $\mu_{v_{2}}$, respectively), and aircraft flight trajectories configuration, and it permits us to state that the set of the parameters used in this paper allows one to realize the conflict point distribution under uncertainties more accurately, which is very important in aviation in terms of safety and effectiveness.
(4) Durind the second comparison of the proposed stochastic model results with the results of Babak et al. [17], it was noticed that the produced method in this paper allowed to achieve slightly smaller random variables combination probability for value 0.95 and the probability ratio $P_{ratio}$ ≈ 0.9 Babak, et al. [17] value.

For future research, the proposed mathematical model investigation of aircraft conflict points and its stochastic distribution under uncertainties could be further investigated. The proposed methodology in this article could be expanded and incorporated together with the authors [32] article where aircraft based on the same input parameters flew via the Dubins and the 3HC methods aiming to resolve conflict situation (at $R_{O_{s}}$ point) though without wind impact. In such proposed incorporated case, aircraft would fly via the Dubins or the 3HC method when the conflict point $(R_{O_{s}}^{\prime }$) would be stochastically distributed.

## Figures and Tables

**Table 1:** Input parameters for *Case 1*, *Case 2*, and *Case 3* stochastic distribution determination.

***Case***	$v_{w}$	$\alpha_{w}$	$\delta_{\alpha_{w}}$	$v_{1}\;$, $v_{2}$
***1***	5, 10, 15, 20, 25 m/s	$\mu_{\alpha_{w}}$ = 0°	±13°	$\mu_{v_{1}}$$=\mu_{v_{2}}$ = 500 kt ($\delta_{v_{1}}$$=\delta_{v_{2}}$ = ±6 kt)
***2***			±20°	
***3***			±26°	

**Table 2:** The $\Delta R_{max}$ conflict point displacement values for *Case 1*, *Case 2*, and *Case 3*.

***Case***	$\Delta R_{max}$ (nm)	5 m/s	10 m/s	15 m/s	20 m/s	25 m/s
***1***	$\Delta R_{max}$	2.186	4.627	7.333	10.304	13.540
***2***		2.487	5.148	7.980	10.987	14.270
***3***		2.660	5.390	8.271	11.281	14.421
